# Genomic Differentiation and Demographic Histories of Two Closely Related Salicaceae Species

**DOI:** 10.3389/fpls.2022.911467

**Published:** 2022-06-07

**Authors:** Zhe Hou, Ang Li

**Affiliations:** College of Landscape Engineering, SuZhou Polytechnic Institute of Agriculture, Suzhou, China

**Keywords:** whole-genome sequencing, genetic diversity, demographic history, adaptation, natural selection

## Abstract

*Populus alba* (*P. alba*) and *Populus davidiana* (*P. davidiana*) are important plant species for answering a variety of issues on species evolution due to their wide distribution and ability to adapt to a variety of environments and climates. Even though *P. alba* and *P. davidiana* belong to ecologically and economically important forest trees in the Northern Hemisphere, little is known about their genomic landscape and genome divergence during speciation. We re-sequenced 20 and 19 members of *P. davidiana* and *P. alba*, respectively, and found that the Dxy value between *P. alba* and *P. davidiana* was 0.2658, whereas the *F*_*ST*_ values were 0.2988, indicating that the genetic divergence was fairly clear. *Populus davidiana* and *P. alba* diverged from the ancestor in the middle Pleistocene, c. 0.80 Ma (95% HPD: 0.79–0.81 Ma). The population sizes of *P. davidiana* increased ~20,000 years ago after a considerable long-term decline following divergence. However, after differentiation, the effective population size of *P. alba* expanded slightly before experiencing a long-term bottleneck effect. According to the expectation of allopatric speciation, we found a significant number of genomic differentiation sites in both species' speciation events, and the majority of these genomic differentiation regions can be attributed to neutral evolutionary processes. Nevertheless, the regions with extreme divergence exist in abundance, indicating that natural selection has had an impact. Positive selection can be found in highly differentiated regions, while long-term balancing selection traits can be easily observed in low differentiated regions. According to these findings, climate differences over the Quaternary, as well as variance in linked selection and recombination, all contributed significantly to genomic divergence during allopatric speciation of the two aspens.

## Introduction

One of the fundamental goals of evolutionary genomics is to understand the relative contributions of diverse evolutionary factors in the generation and shaping of genetic diversity within and among species (Nordborg et al., 2005). Determining the evolutionary forces affecting patterns of genome-wide variation has been a central goal in evolutionary biology over the past several decades (Seehausen et al., 2014). An important aspect of understanding speciation is the study of variation in levels of differentiation among closely related species (Mark et al., 2018). Under strict neutrality, differentiation is expected to accumulate as a result of the stochastic fixation of polymorphisms by genetic drift (Hellmann et al., 2005). Demographic fluctuations, including population expansions and bottlenecks, can decelerate or accelerate the rate of differentiation and genome-wide variation in various ways (Li and Durbin, 2011). Historical, geographic and climatic events can trigger population differentiation by affecting the demographic history of the populations (Sanna et al., 2008). In the absence of gene flow, differentiation between populations increases gradually as a result of natural selection and/or random processes like mutation and genetic drift (Begun et al., 2007).

For those genome regions that are affected by natural selection, the linked sites can also be affected in several ways (Via, 2009). Moreover, as the speciation process progresses, numerous forms of selection can impact the patterns of development of genomic divergence and genetic variation. Various types of natural selection (purifying, balancing, and positive selection) are adequate in these settings to produce varied genomic differentiation patterns and even speciation (Turner et al., 2005; Noor and Bennett, 2009). Regardless of the role of natural selection, demographic dynamics, stochastic genetic drift, and recombination rate can all alter genome-wide patterns of diversity and genomic architecture of adaptation (Nosil et al., 2009; Campagna et al., 2015). The hypotheses outlined previously are generally not mutually exclusive, and a complete analysis of these hypotheses requires a thorough understanding of the speciation process, such as the period of speciation and the demographic and geographic environment in which it occurred (Nosil and Feder, 2012). As it becomes increasingly feasible to generate whole genome resequencing data from closely related species, the importance of conserved genomic features in shaping the topography of the genomic landscape of speciation has increasingly been highlighted (Wang et al., 2020).

Forest trees provide a promising resource to address adaptive evolution and patterns of genome variation because most of them are undomesticated, possess a variety of phenotypic and genetic variations, and can adapt to a variety of climates (Neale and Antoine, 2011). Here, we focus on two *Populus* species (*Populus alba* L and *Populus davidiana* Dode) to evaluate the evolutionary forces affecting patterns of genome-wide variation. The two species are all from the section *Leuce Duby* of the genus *Populus*. *Populus alba* and *P. davidiana* are the most ecologically important forest trees in China, as well as ecologically important members of mesic forest ecosystems that were isolated by Pleistocene glaciations (Jianchao et al., 2018). Initial investigations based on complete plastid genomic data (Lei et al., 2018) and resequenced genomes (Wang et al., 2020) demonstrated that *P. alba* and *P. davidiana* have the closest relationship. *Populus davidiana* and *P. alba* are sibling aspen species based on their close phylogenetic relationship and morphological similarity belonging to the section of *Leuce* (*Populus*) (Eckenwalder, 1977). *Populus davidiana* in this study is mainly distributed in the northeastern part of China, which is very cold, but *P. davidiana* is a strong positive species, tolerant of cold and dry and infertile soil (Zong et al., 2019). *Populus alba* is mainly distributed in the Xinjiang region of China, which has a very arid climate and little rainfall, but *P. alba* is wind-resistant and tolerant of dry climate (Honglei et al., 2017). The taxonomy of these two aspens has been controversial with respect to their extreme morphological congruence with only minor differences in leaf shape (Löve and Löve, 1975). However, they have significant genetic and ecological differences, indicating that a strong divergent ecological selection has influenced them (Honglei et al., 2017). *Populus alba* and *P. davidiana* have close genetic affinity based on previous phylogenetic analyses (Supplementary Figure 2) (Liu et al., 2016). *Populus alba* and *P. davidiana* are sister species, according to a recent study (Zong et al., 2019) based on morphological analysis and whole chloroplast genome sequences (Supplementary Figure 3).

Both *P. davidiana* and *P. alba* have wide geographic distribution, high intraspecific polymorphism, adaptability to different environments, phenotypic diversity, combined with a relatively small genome size (Liu et al., 2019), and further facilitation has taken from the accessibility of reference data of a good caliber from the genome of *Populus trichocarpa* Torr. & A. Gray ex. Hook. reference genome, version 3 (Tuskan, 2006), the two species provide an ideal system to study heterogeneous genomic differentiation, genetic diversity, demographic history, adaptation, and gene flow during the process of speciation. In the current work, we collected and sequenced 39 individuals from *P. davidiana* and *P. alba* populations in China. We specifically aimed to (i) explore their population structure; (ii) estimate the historical demographic processes and species divergence time points; (iii) infer the overall patterns of heterogeneous genomic differentiation and fine-scale genomic landscapes of diversity; (iv) identify signatures of long-term balancing selection and positive selection over the entire genome. Specifically, the main purpose of this study is to understand and disentangle how the patterns of genetic variation are shaped by the multitude of evolutionary forces within and among species during the process of speciation.

## Materials and Methods

### Population Sample Collection, Sequencing, and Genotype Calling

Silica-gel dried leaves of 19 *P. alba* and 20 *P. davidiana* individuals covering their geographical distributions in China were collected for DNA extraction (Table 1). Genomic DNA from the leave of each individual was extracted using the CTAB method (Pahlich and Gerlitz, 1980) and Qubit dsDNA BR assay (Life Technologies, Carlsbad, CA, USA) was employed to assess the quantity and quality of DNA. Paired-end sequencing libraries with an insert size of 600 bp were constructed using the standard Illumina HiSeq 2000 platform protocol. Later on, every *P. alba* and *P. davidiana* specimen was sequenced on the Illumina HiSeq 2000 platform. All samples were sequenced to a target coverage of 30×. The raw sequence data reported in this work have been submitted with the Genome Sequence Archive (Wang et al., 2017) at the BIG Data Center, Beijing Institute of Genomics (BIG), Chinese Academy of Sciences, with accession numbers CRA003302 and CRA001674 for samples of *P. alba* and *P. davidiana*, respectively. CRA003302 and CRA001674 are publicly accessible at http://bigd.big.ac.cn/gsa. Adapter sequences of raw sequencing reads were eliminated with Trimmomatic (Lohse et al., 2012) before reading mapping. The bases were cut off and trimmed from the beginning or the ending of reads when the base quality was ≤20. Reads were completely discarded if there were fewer than 36 bases remaining after trimming. The remaining reads were mapped via the BWA-MEM algorithm employing the default parameters in BWA v0.7.8 (Li et al., 2009) against *Populus trichocarpa* Torr. & A. Gray ex. Hook. reference genome, version 3 (Tuskan, 2006). We further employed several filtering steps to minimize the influence of mapping bias before genotype calling. First, the genome regions around deletions or insertions had some misalignment of bases, and we corrected the bias by IndelRealigner and RealignerTargetCreator in GATK v3.8.0 (Depristo et al., 2011) with default parameters. In order to account for the occurrence of PCR duplicates introduced during library construction, we used MarkDuplicates in Picard (http://picard.sourceforge.net) to remove reads with identical external coordinates and insert lengths. For downstream analyses, we only retained the reads with the highest summed base quality. We further filtered the reads that could potentially cause mapping bias according to three additional standards: (1) those with extreme read coverage (<4 or higher than twice of the mean coverage); (2) RepeatMasker detecting overlapping known repeated elements (Tarailo-Graovac and Chen, 2009); (3) covered by greater than two reads of mapping score equaling zero per individual.

**Table 1:** Statistics summary of Illumina re-sequencing data per sample.

**SampleID**	**Location**	**Latitude**	**Longitude**	**Mapping**	**Data**
				**rate (%)**	**volume(Gb)**
***P. davidiana***					
*P. davidiana*1	Shuangyashan	E 131°15′	N 46°64′	92.36	6.8
*P. davidiana*2	Shuangyashan	E 131°15′	N 46°64′	91.25	6.5
*P. davidiana*3	Huanan	E 130°55′	N 46°23′	92.08	8.2
*P. davidiana*4	Huanan	E 130°55′	N 46°23′	91.78	6.3
*P. davidiana*5	Heihe	E 127°52′	N 50°24′	91.25	6.9
*P. davidiana*6	Heihe	E 127°52′	N 50°24′	92.36	6.1
*P. davidiana*7	Heihe	E 127°52′	N 50°24′	92.08	6.2
*P. davidiana*8	Heihe	E 127°52′	N 50°24′	91.98	6.6
*P. davidiana*9	Heihe	E 127°52′	N 50°24′	92.05	7.0
*P. davidiana*10	Lianhuashan	E 124°49′	N 44°47′	93.25	7.2
*P. davidiana*11	Lianhuashan	E 124°49′	N 44°47′	91.28	6.8
*P. davidiana*12	Lianhuashan	E 124°49′	N 44°47′	91.89	6.3
*P. davidiana*13	Lianhuashan	E 124°49′	N 44°47′	91.21	7.3
*P. davidiana*14	Shuangshan	E 123°88′	N 43°68′	93.25	6.0
*P. davidiana*15	Shuangshan	E 123°88′	N 43°68′	92.08	6.1
*P. davidiana*16	Weichang	E 117°76′	N 41°93′	91.23	6.4
*P. davidiana*17	Wutai	E 113°25′	N 38°72′	91.78	7.5
*P. davidiana*18	Hualin	E 116°40′	N 39°90′	91.96	7.1
*P. davidiana*19	Wutai	E 113°25′	N 38°72′	91.98	6.9
*P. davidiana*20	Hualin	E 116°40′	N 39°90′	92.25	6.2
***P. alba***					
*P. alba*1	Xinjiang	E 131°15′	N 46°64′	92.36	5.9
*P. alba*2	Xinjiang	E 131°15′	N 46°64′	91.25	6.2
*P. alba*3	Xinjiang	E 130°55′	N 46°23′	92.08	7.3
*P. alba*4	Xinjiang	E 130°55′	N 46°23′	92.78	6.3
*P. alba*5	Xinjiang	E 127°52′	N 50°24′	91.25	6.9
*P. alba*6	Xinjiang	E 127°52′	N 50°24′	92.36	6.2
*P. alba*7	Xinjiang	E 127°52′	N 50°24′	91.08	6.1
*P. alba*8	Xinjiang	E 127°52′	N 50°24′	91.98	6.1
*P. alba*9	Xinjiang	E 127°52′	N 50°24′	92.05	6.8
*P. alba*10	Xinjiang	E 124°49′	N 44°47′	93.25	6.5
*P. alba*11	Xinjiang	E 124°49′	N 44°47′	91.28	6.7
*P. alba*12	Xinjiang	E 124°49′	N 44°47′	91.89	6.0
*P. alba*13	Xinjiang	E 124°49′	N 44°47′	91.21	6.9
*P. alba*14	Xinjiang	E 123°88′	N 43°68′	93.25	6.6
*P. alba*15	Xinjiang	E 123°88′	N 43°68′	92.08	6.2
*P. alba*16	Xinjiang	E 123°88′	N 43°68′	91.78	6.0
*P. alba*17	Xinjiang	E 123°88′	N 43°68′	92.58	6.3
*P. alba*18	Xinjiang	E 126°66′	N 51°72′	91.32	6.5
*P. alba*19	Xinjiang	E 126°66′	N 51°72′	93.25	6.0

After filtering, two complementary methods were implemented for downstream analysis. First, a series of population genetic analyses was carried out using ANGSD v0.917. The pipeline of ANGSD v0.917 relies on allele frequency spectrum and genotype probabilities but does not take genotype calls into account (Korneliussen et al., 2014). Second, the HaplotypeCaller of the GATK v3.8.0 was implemented to perform genotype calling in each individual for downstream analysis requiring accurate genotype calls. For re-annotation and re-genotyping of the newly merged VCF, we used GenotypeGVCFs to unify multi-sample records from the two species together (Depristo et al., 2011). We further performed a set of filtering steps to reduce genotype calling bias, such that the downstream analyses were carried out by retaining only high-quality single nucleotide polymorphisms (SNPs): (1) removed all SNPs sites that had not passed previously the filtering criterion; (2) reserved only 2 alleles with a distance ≥5 bp away from any indels; (3) genotypes with read depth (DP) <5 or with a genotype quality score (GQ) <10 were categorized as missing, all SNPs with a genotype missing rate over 10% were discarded.

### Population Structure, Genetic Relationship, and Principal Components Analysis

NGSadmix was implemented to deduce the genetic structure of the *P. alba* and *P. davidiana* population, and only used sites with ≤10% of missing data (Skotte et al., 2013). The SAMTools model in ANGSD (Li et al., 2009) was employed to assess the genotype likelihoods and later a beagle file was generated to infer the population genetic structure with a likelihood ratio test (*P* < 10^−6^) (Wang et al., 2016), and the count of genetic clusters (K) ranging from 1 to 6. The program of smartpca in PCAngsd software (http://www.popgen.dk/software/index.php/PCAngsd) was used to perform the Principal component analysis (PCA) with a Tracy–Widom test to ascertain the significance level of eigenvectors. The neighbor-joining (NJ) model with TreeBest software (http://treesoft.sourceforge.net/treebest.shtml) was used for the construction of the phylogenetic tree.

### Demographic History

We used individuals with high sequencing coverage (>35×) from each population and applied the Pairwise Sequentially Markovian Coalescent (PSMC) model to reconstruct demographic history (Li and Durbin, 2011). The parameter was set to the default parameter during the calculating procedure. Based on these parameters to covert scaled population sizes and time to actual sizes and time, the mutation rate was set as 3.75 × 10^−8^ per base per generation, and we adopted a generation time of 15 years. We carried out 100 bootstrapping simulations to estimate the variance fluctuation of population size (Koch et al., 2000).

We further used Fastsimcoal 2.6.1 software (Excoffier et al., 2013) to infer past demographic histories, the divergence time, and gene flow patterns of *P. alba* and *P. davidiana*. Allele frequencies in the 39 samples were calculated using the realSFS module in ngsTools software so as to construct the required two-dimensional joint site frequency spectrum (2D-SFS), which was estimated with 100,000 coalescent simulations in each model. Alternative models of historical events were fitted to the joint site frequency spectra data (Fumagalli et al., 2014). We simulated 18 different models and all these models proceed with the divergence of an ancestral species into *P. alba* and *P. davidiana* lineage. Various models were made to model the earlier demographic and speciation histories of *P. alba* and *P. davidiana* that varied with respect to (1) No Migration and no population expansion; (2) Asymmetric Migration but no population expansion; (3) Asymmetric Migration along with population expansion; (4) complicated model, comprising a bottleneck in *P. alba*; (5) complicated model, containing a bottleneck in *P. davidiana* (Supplementary Figure 1). The two-dimensional joint SFS data had a good fit with the alternative demographic history models. We obtained global maximum likelihood estimates for the 18 different models from 50 individual runs, with 40 conditional rounds of the likelihood maximization algorithm. The criteria for comparison of these models was the maximal value of likelihood generated by 50 independent runs on the basis of calculated Akaike's weight (Excoffier et al., 2013). The model with the highest Akaike's weight estimate was deemed the best of the models. Parametric bootstrapping was carried out with 100 bootstrap cycles and 50 independent runs in every single bootstrap and was used to calculate confidence intervals. We also considered 15 years as a generation (Tuskan, 2006) and the mutation rate of each generation was 3.75 × 10^−8^ (Ingvarsson, 2008) for the conversion of estimates to units of individuals and years.

### Genetic Diversity and Divergence

The software VCFtools with the default parameters was used to calculate the levels of genetic differentiation (Weir and Cockerham mean *F*_*ST*_), nucleotide diversity (π), and Tajima's D for individual species. We then took an average of (within each 10 Kbp non-overlapping window) *F*__*ST*__, π, and Tajima's D values of all sites (Petr et al., 2011).

### Genome-Wide Patterns of Differentiation and Detection in Outlier Windows

In order to examine thresholds for detection of outlier windows that may have been targets of natural selection, we conducted coalescent simulations to compare observed patterns of genetic differentiation (*F*ST) to those expected under different demographic models (see Results). All simulations were performed using the program *msms* v3.2rc based on demographic parameters derived from the best-fitting model inferred by *fastsimcoal2.5.1*. Population-scaled recombination rates ρ were assumed to be between 1 and 5 Kbp^−1^ given the large variation. We simulated genotypes corresponding to a 10 Kbp region with the same sample size as the real data for 100,000 replications, from where we simulate genotype likelihoods using the program msToGlf in ANGSD (Korneliussen et al., 2014) by assuming a mean sequencing depth of 20× and an error rate of 0.5%. We estimated two summary statistics, nucleotide diversity π and Tajima's D, from sample allele frequency likelihoods in ANGSD for all simulation replicates to test whether the simulated data matches the observed data. To assess whether any of the observed windows display *F*ST values deviating significantly from neutral expectations, we determined the conditional probability (*P*-value) of observing more extreme inter-specific *F*ST values among simulated data sets than among the observed data. The determination of significance was based on running 500,000 coalescent simulations of the most acceptable demographic null model. We then corrected for multiple testing by using the False Discovery Rate (FDR) adjustment, and only windows with FDR lower than 1% were considered as candidate regions affected by selection.

### Population Genetic Analysis and Molecular Signatures in Outlier Regions

In both *P. alba* and *P. davidiana* species, multiple population genetic analyses were contrasted with the remaining regions of the genome in order to infer the molecular signatures of selection in outlier regions manifesting either extremely low or high degree of differentiation. ANGSD was first employed to assess the probabilities of sample allele frequency between populations of the *P. alba* and *P. davidiana* over non-overlapping 10 Kbp windows for the estimation of θπ, Fu and Li (1993) and Fay and Wu (2000). Further, to calculate the correlation coefficients (*r*^2^) for evaluating the levels of LD within each 10 Kbp window, we used VCFtools v0.1.12b (Danecek et al., 2011). The recombination rates (ρ) within each 10 Kbp window were calculated using FastEPRR software (Gao et al., 2016). Finally, ngsStat (Fumagalli et al., 2014) was used to estimate a number of additional population genetic parameters of genetic differentiation: (1) the proportion of derived alleles fixed in either *P. alba* and *P. davidiana* and the proportionate number of inter-specific shared polymorphisms between all segregating sites; (2) dividing the dxy of the *P. alba* and *P. davidiana* species by the dxy between the *P. alba* population and *P. tremula* to estimate the relative node depth (RND); (3) the posterior probability of the sample allele frequency at each locus and averaged over each 10 Kbp window was used to calculate dxy. Wilcoxon ranked-sum tests were used to analyze the genome-wide mean for all population genetic data presented, as well as the significance of differences among outlier windows.

### Gene Ontology (GO) and KEGG Pathways Enrichment

GO enrichment analysis was used to test if the highly differentiated regions had any overrepresentation of functional classes of genes, and a Fisher's exact test was carried out using agriGO's Term Enrichment tool (http://bioiSo.cau.edu.cn/agriGO/index.php) (Du et al., 2010). In an attempt to rectify the *P*-value corresponding to Fisher's exact test, numerous tests were carried out by employing the Benjamini-Hochberg error detection rate. Taking *P*-value under 0.05 as standard, we identified significantly enriched GO terms. The KEGG pathways were analyzed using the KOBAS system (Mao et al., 2005) and the FDR method was applied to correct the different comparisons.

## Results

We generated whole-genome resequencing data for 19 *P. alba* and 20 *P. davidiana*. *Populus alba* and *P. davidiana* were found to have a high degree of conservation in their genomes (Pakull et al., 2009), in a way that roughly 91.08% (Table 1) of all sequences of *P. alba* and *P. davidiana* can be conveniently mapped to the *P. trichocarpa* reference genome (Tuskan, 2006) following a quality control process. In the mapped reads of *P. alba* and *P. davidiana* samples, the mean coverage of individual site reached 31.86 (Table 1). SNP and genotype data were reliably obtained by two different yet complementary methods: (1) High-quality site-frequency-spectrum (SFS) data were obtained using ANGSD software (Korneliussen et al., 2014) for determining the genetic parameters of the population without calling genotypes (Nielsen, 2005). (2) Multi-sample SNP and genotype calling was implemented in GATK v3.2.2 with HaplotypeCaller and GenotypeGVCFs (Danecek et al., 2011). Following filtration and thorough quality control, the 19 *P. alba* and 20 *P. davidiana* samples yielded 5,035,016 and 5,158,369 high-quality SNP sites, respectively.

### Structure of the Population

**Figure 1:**
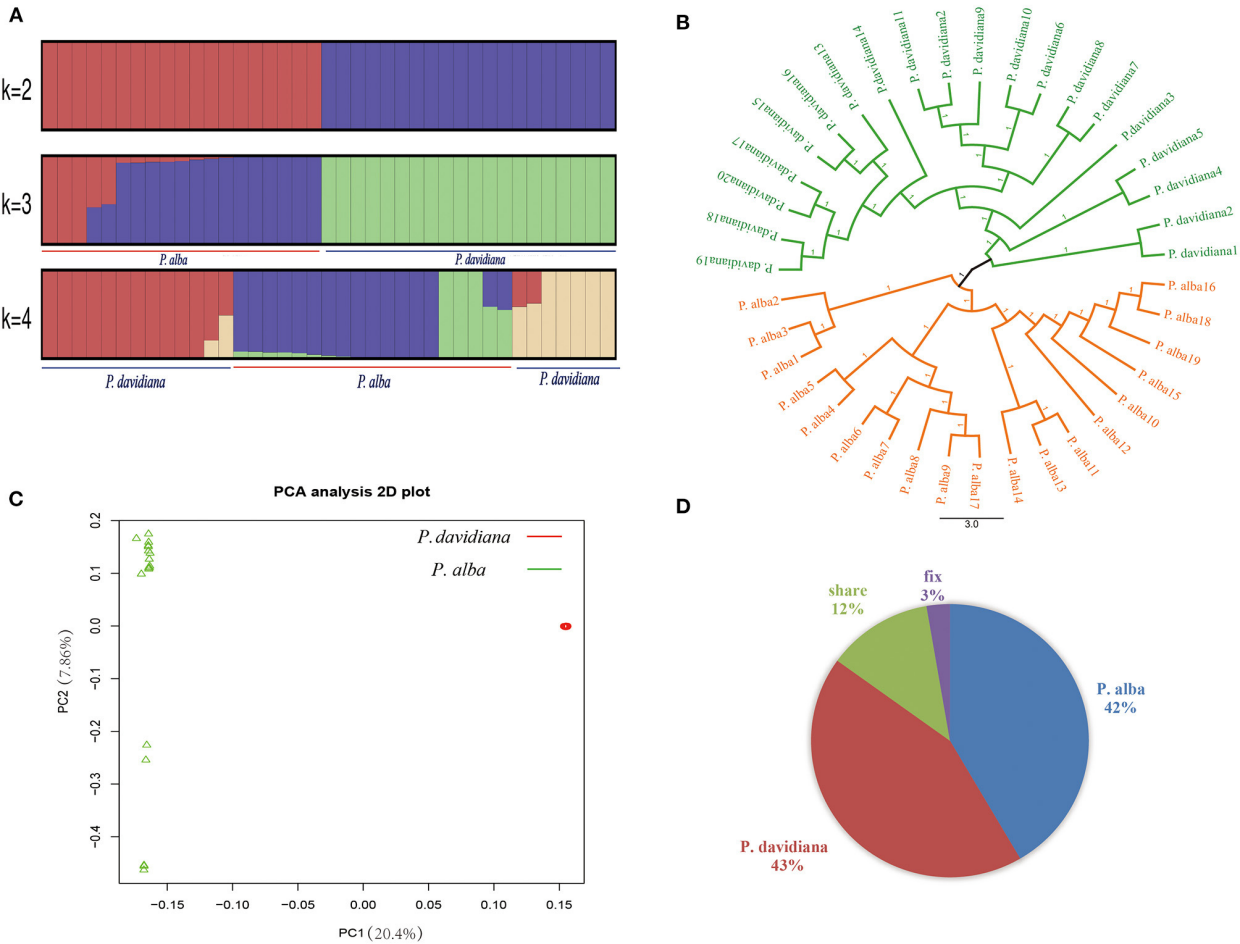
Genetic structure of 20 *Populus davidiana* and 19 *Populus alba*. **(A)** Genetic structure of *P. davidiana* and *P. alba* inferred using NGSadmix. **(B)** A rooted neighbor-joining tree constructed from the allele-shared matrix of SNPs among the *P. davidiana* and *P. alba*. **(C)** Principal component analysis (PCA) plot based on genetic covariance. **(D)** Pie chart summarizing the proportion of fixed, shared and exclusive polymorphisms of the two species.

NGSadmix was employed to infer individual ancestry based on genotype likelihoods, which takes the uncertainty of genotype calling into account. When K was set as 2, the majority of the members were categorized into two different genetic clusters. When *K* = 3, *P. alba* showed additional sub-structuring of the population, but the bulk of *P. alba* members were deduced to be a product of two genetic components mixing, demonstrating very minor clinal variation with increasing latitude. When *K* = 4, hybridization and introgression can be found between *P. alba* and *P. davidiana* (Figure 1A). We constructed a neighbor-joining tree that further reinforced these patterns, with the different geographical placement of *P. alba* and *P. davidiana* reflecting the grouping of populations (Figure 1B). The results were also reinforced by PCA since there were two distinct populations of the 39 *P. alba* and *P. davidiana* specimens taken from different regions (Figure 1C). Fixed differences accounted for 3% of polymorphisms in the two populations, whereas shared polymorphism accounted for 12%, and private polymorphic loci accounted for 42% and 43% polymorphism in the populations of *P. alba* and *P. davidiana*, respectively (Figure 1D).

### Demographic Histories

**Figure 2:**
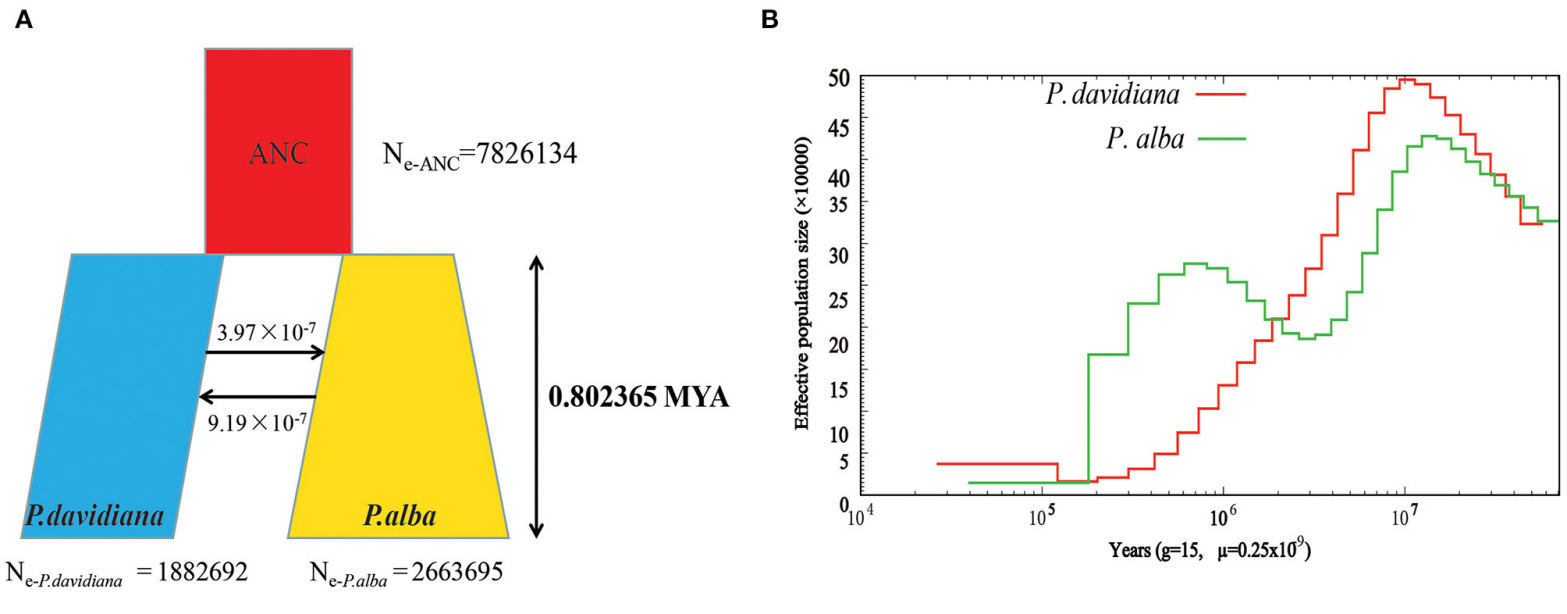
**(A)** Best-fitting model inferred demographic histories and differentiation mode for *Populus davidiana* and *P. alba* implemented by fastsimcoal 2.6.1. **(B)** The effective population size (Ne) over historical time implementing by PSMC.

The demographic histories and timings of divergence of the two species were further estimated by employing a coalescent simulation-based technique carried out in Fastsimcoal 2.6.1 (Excoffier et al., 2013). There were 18 models developed that focused on the divergence of *P. alba* and *P. davidiana* (Supplementary Figure 1). A comparison of the AIC values for all four scenarios established that the complex isolation-with-migration model (model 4; Figure 2A; Table 2) with the highest likelihood and lowest AIC value is the best-fitting model. As revealed by the estimates of parameter acquired from the best model, it was found that following the divergence of the two species, a stepwise change in the population size occurred in *P. davidiana*, however *P. alba* underwent exponential growth (Figure 2A). Table 3 lists the details regarding differentiation time point, effective population size, and gene flow of *P. alba* and *P. davidiana*, displaying the 95% confidence interval (CIs) for the associated variables alongside. Around 0.80 million years ago (Mya), the progenitors of the two aspens underwent divergence into *P. alba* and *P. davidiana* populations (bootstrap range [BR]: 0.79–0.81 Mya). Currently, the functional population sizes (Ne) of *P. davidiana* (N_e−__*P*.*davidiana*_) and *P. alba* (N_e−__*P*.*alba*_) are 1,882,692 (BR:1,863,658–1,891,256) and 2,663,695 (BR: 2,651,235–2,678,958) respectively. However, both species have a significantly low effective population sizes in comparison to their common ancestor (N_e−ANC_ = 7,826,134 [7,652,251–7,962,365]). The rate of migration (m) also distinguishes between the two species, the least generation migration rate (m) between *P. alba* and *P. davidiana* (3.97 × 10^−8^ and 9.16 × 10^−7^), which is not accidental as the two populations are separated by a significant geographical distance.

**Table 2:** Relative likelihood of the different models.

**Model**	**Max(log10(Lhoodi)^a^**	** ${\text{AIC}}_{\text{i}}^{\text{b}}$ **	**Δi^b^**	**Model normalized**
				**relative likelihood**
				**(w_**i**_)^**b**^**
Model 1	−752381556.3	356121231.2	3569618.436	~0
Model 2	−766668258.6	355678656.1	3658352.526	~0
Model 3	−769356470.5	354588633.5	2636451.235	~0
Model 4	−707411635.8	356531580.6	0	~1
Model 5	−738200638.82	355259547.5	2065856.026	~0
Model 6	−742253556.3	355955172.2	216486.022	~0
Model 7	−742377887.6	358863315.6	1868753.036	~0
Model 8	−741803292.7	354568532.2	658541.035	~0
Model 9	−72078163.5	353426226.1	1522688.015	~0
Model 10	−737524429.5	353223549.6	1478223.020	~0
Model 11	−737955129.5	352793852.6	4583631.025	~0
Model 12	−740129825.3	353659253.0	2587456.015	~0
Model 13	−743961354.6	353336542.2	5214852.061	~0
Model 14	−743705455.5	356823658.2	136586.099	~0
Model 15	−741996176.5	353338885.3	1253589.018	~0
Model 16	−740495214.920	356852515.6	2659565.498	~0
Model 17	−745052395.561	359596326.3	2568742.259	~0
Model 18	−739893331.173	355944120.3	375961.568	~0

a *Based on the best likelihood among the 50 independent runs for each model*.

b *The calculation of AIC_i_, Δ_i_, and w_i_ are according to the methods shown in Excoffier et al. (2013)*.

**Table 3:** Demographic parameters and confidence interval of the best model.

	**Point estimation**	**95% CI^a^**	
		**Lower**	**Upper**
**Parameters**		**bound**	**bound**
N_e−ANC_	7826134	7652251	7962365
N_e−P.davidiana_	1882692	1863658	1891256
N_e−P.alba_	2663695	2651235	2678958
m_P.davidiana−>**P*.*alba**_	3.97 ×10^−7^	3.50 ×10^−7^	4.15 ×10^−7^
m_P.alba−>**P*.*davidiana**_	9.19 ×10^−7^	8.99 ×10^−7^	9.98 ×10^−7^
TDIV	802365	793658	812546

*Parameters are defined in Figure 2A. N_e−ANC_, N_e−N_, N_e−P.davidiana_, and N_e−P.alba_ indicate the effective population sizes of ancestral, P. davidiana and P. alba population respectively, m_P.davidiana−>P.alba_ indicates the per generation migration rate from P. davidiana to P.alba, m_P.alba−>P.davidiana_ indicates the per generation migration rate from P.alba to P. davidiana, TDIV indicates the estimated divergence time between P. davidiana and P. alba populations*.

a *Parametric bootstrap estimates obtained by parameter estimation from 100 data sets simulated according to the overall maximum composite likelihood estimates shown in point estimation columns. Estimations were obtained from 100,000 simulations per likelihood*.

PSMC (Li and Durbin, 2011) was used to estimate demographic history which depicted that a dramatic decline was experienced by both species in terms of population size around 100 Ma (Figure 2B). In comparison to the population of *P. alba*, which only recovered moderately about 800 kya, the population sizes of *P. davidiana* increased around 20,000 years ago, after a long period of decrease following divergence (Figure 2B).

### Genome Differentiation and Identification of Outlier Regions

We have previously shown that linkage disequilibrium (LD) decays within 10 kilobases (Kbp) in both *P. alba* and *P. davidiana*, and thus 10,000 bp windows were used to investigate patterns of genomic differentiation.between species. As a standard parameter evaluating genetic differentiation, the fixation index *F*_*ST*_ is sensitive to any event or process that leads to an alteration in interspecific variation (Cruickshank and Hahn, 2014). The current work involves the calculation of the genetic differentiation coefficient *F*_*ST*_ for the two species. Since the mean *F*_*ST*_ value between the two species was 0.2988 (**Table 5**), genetic differentiation was found to be evident among the two populations. Total sequence differentiation among the populations which stands as a universal criterion for evaluating interspecific differentiation and is referred to as dxy was also calculated. The two populations were found to have evident sequence differentiation among them, with the mean value of dxy between *P. davidiana* and *P. alba* being 0.2658 (**Table 5** and Supplementary Table 1).

Comparing the empirical distribution of inter-specific *F*_*ST*_ with that obtained from simulations based on the best-fitting demographic model, we identified 379 and 1,156 outlier windows exhibiting significantly (False Discovery Rate <0.01) high and low interspecific *F*_*ST*_ compared to the expected null distribution obtained from the coalescent simulations (Supplementary Tables 2, 3).

### Population Genetic Analysis

**Figure 3:**
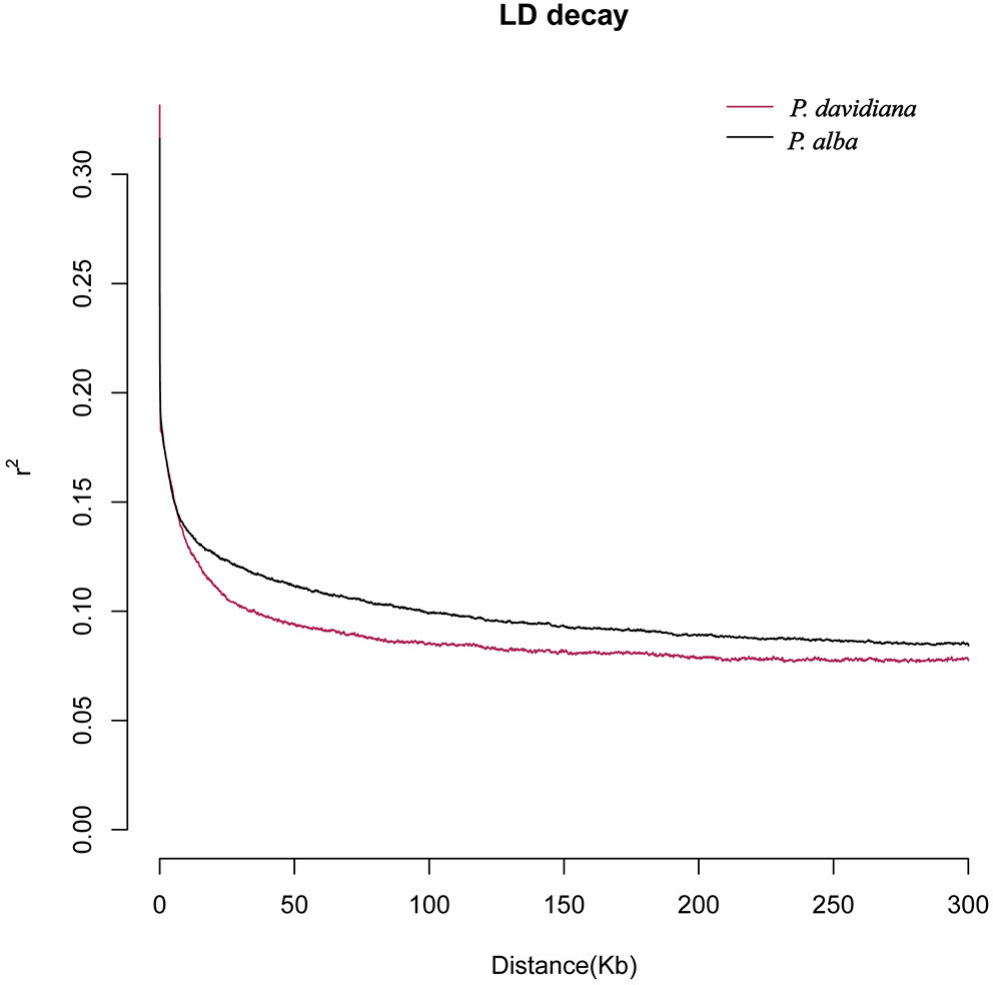
Linkage disequilibrium decay patterns for *P. davidiana* and *P. alba*.

The parameters θπ, θw, H_E_, ρ, and Tajima's D were calculated for the two species to estimate the patterns of genetic diversity throughout the entire genome. The lowest H_E_ was manifested by individuals of *P. davidiana* (**Table 5**), whereas the highest H_E_ was harbored by individuals of *P. alba*. In light of the results of H_E_, relatively lower genetic diversity (π = 0.0089) is exhibited by *P. davidiana* in comparison to *P. alba* (π = 0.0095; **Table 5**). *P. alba* possesses had a slower LD decay and a slightly higher recombination rate (**Table 5**) compared to *P. davidiana* (Figure 3). In comparison to *P. davidiana* (Tajima's D = 0.13), *P. alba* (Tajima's D = −0.06) has a lower Tajima's D-value. The physical distance after the average LD coefficient *r*^2^ decays to half of the maximal value is generally referred to as the LD decay distance. Different LD decay curves are manifested by the *P. alba* and *P. davidiana* populations (Figure 3), suggesting diverse demographic backgrounds of both species, population reduction, or genetic differentiation may alter the LD pattern of the genome. The *P. davidiana* population has the fastest decay rate and the smallest LD value, whereas the *P. alba* population had the slowest decay rate and the largest LD value (Figure 3).

### Signatures of Selection in Outlier Regions

**Figure 4:**
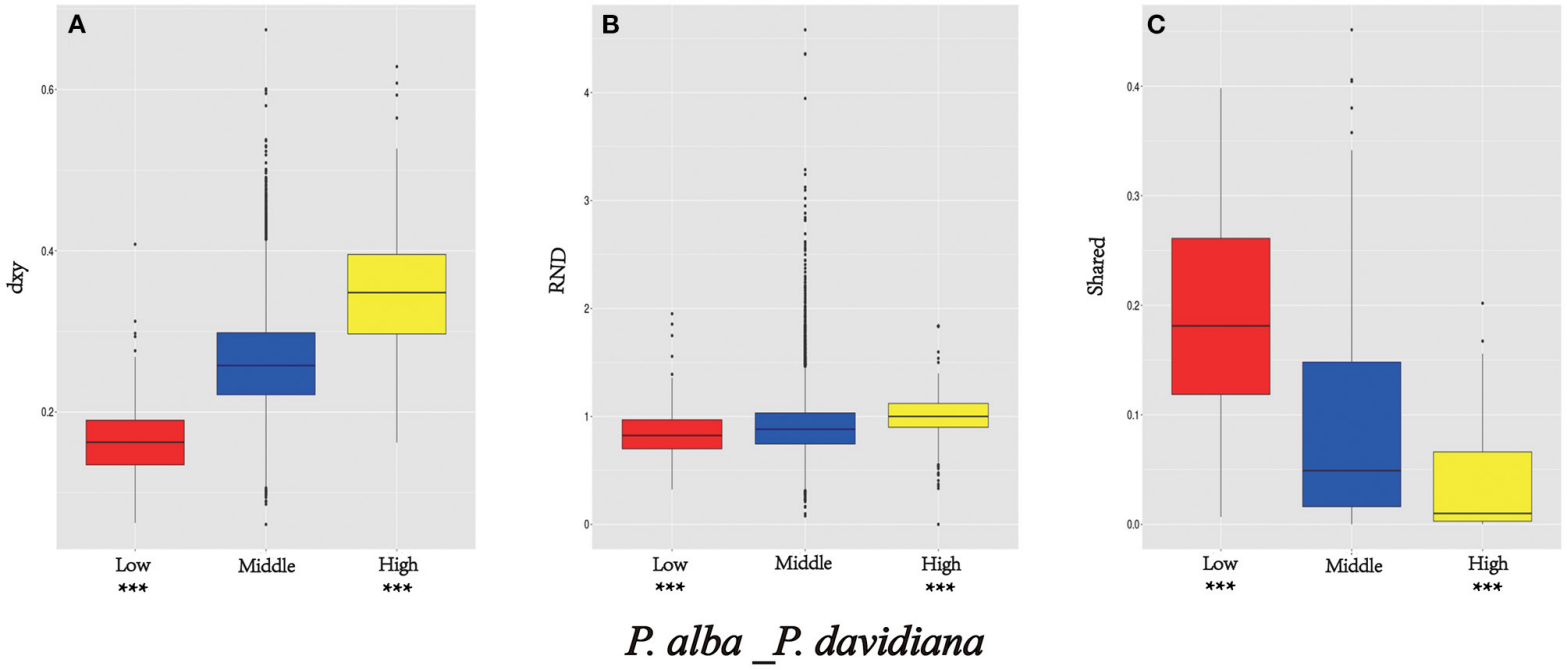
Comparisons of dxy **(A)**, RND **(B)**, and shared **(C)** among regions displaying significantly high (yellow boxes) and low (red boxes) differentiation vs. the genomic background (blue boxes) between *P. davidiana* and *P. alba*. Asterisks designate significant differences between outlier windows and the rest of genomic regions by Mann-Whitney U test (***P-value < 2.2e–16).

**Figure 5:**
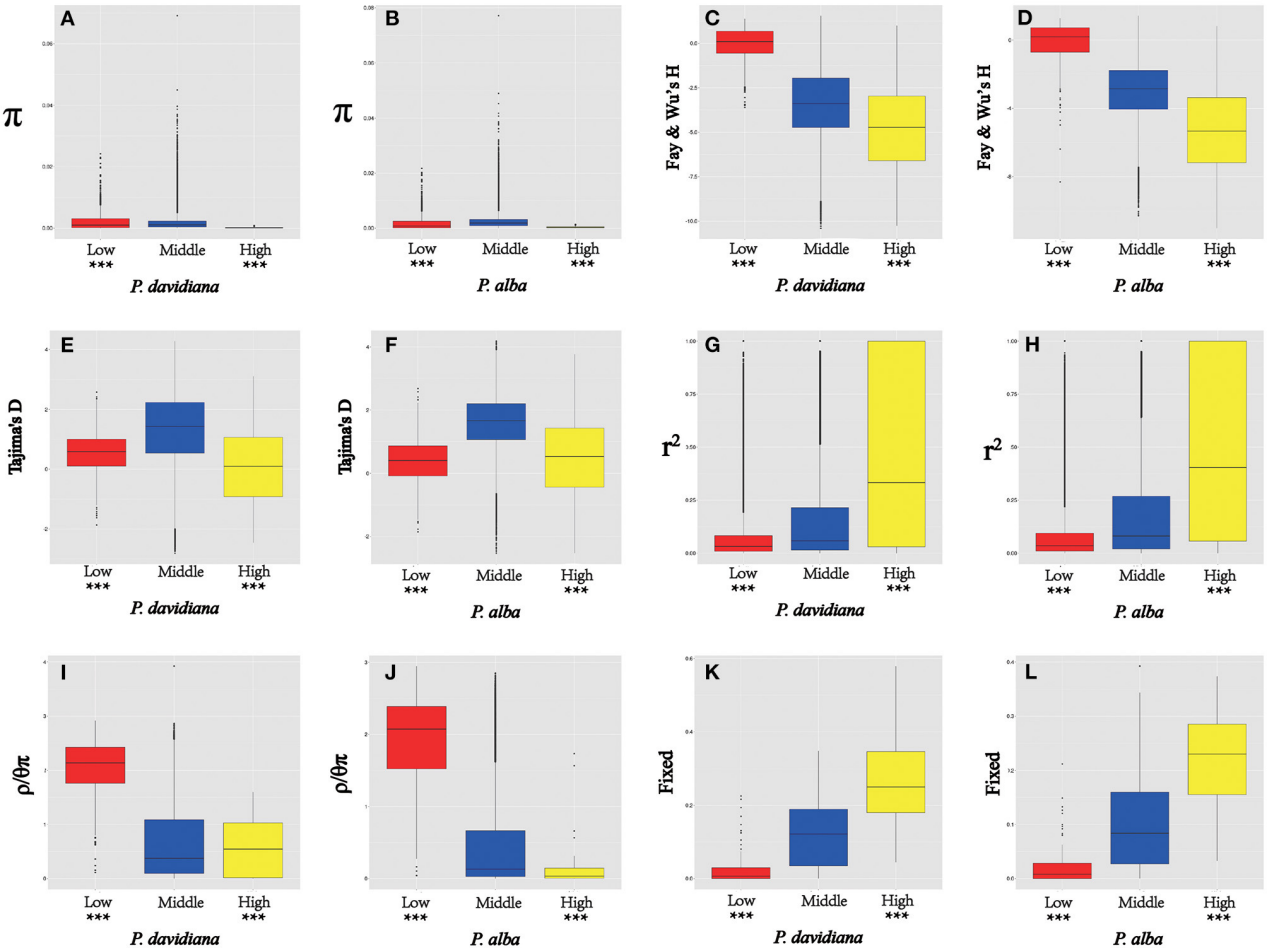
The outlier regions are significantly influenced by natural selection. **(A,B)** Comparisons of nucleotide diversity π among regions displaying significantly high (yellow boxes) and low (red boxes) differentiation vs. the genomic background (blue boxes) between *P. davidiana* and *P. alba*.; **(C,D)** Comparisons of Fay & Wu's H in *P. davidiana* and *P. alba*; **(E,F)** Comparisons of Tajima's D in *P. davidiana* and *P. alba*; **(G,H)** Comparisons of *r*^2^ in *P. davidiana* and *P. alba*; **(I,J)** Comparisons of recombination rate (ρ/θπ) in *P. davidiana* and *P. alba*; **(K,L)** Comparisons of the proportion of fixed differences caused by derived alleles fixed in *P. davidiana* and *P. alba*. Asterisks designate significant differences between outlier windows and the rest of genomic regions by Mann-Whitney U test (***P < 2.2e–16).

As *F*_*ST*_ is a relative measure of differentiation and is thus sensitive to any processes that alter intra-species genetic variation, we quantified and compared inter-specific genetic differentiation between two unions of outlier windows and the rest of the genome using several additional approaches (Table 4). Compared to the genomic background averages, both dxy and RND revealed significantly greater divergence between the two species in regions of high differentiation (**Figures 5A,B**). For both populations, the highly differentiated regions manifested distinct positive selection characteristics (Nielsen, 2005). As an example, both *P. alba* and *P. davidiana* populations had exceptionally low levels of polymorphism (π) (**Figures 5A,B**). The more negative Fay & Wu's H values reflected frequently appearing derived alleles (**Figures 5C,D**), whereas the more negative Tajima's D values reflected frequently appearing rare alleles (**Figures 5E,F**). A more visible characteristic is the regions with high differentiation having powerful signals of linkage disequilibrium (LD) (**Figures 5G,H**) (*P* < 0.001, Mann-Whitney U test). Inter-specific shared polymorphisms among the two populations as well as the alleles fixed in the *P. alba* and *P. davidiana* populations were also compared. The results indicated that in the regions with high differentiation, the ratio of inter-specific shared polymorphisms was very low (Figure 4C) and in both the *P. alba* and *P. davidiana* populations, the proportion of fixed differences was notably high (Figures 5K,L).

**Table 4:** Statistics **s**ummary comparing regions displaying extreme genetic differentiation with the rest of the genomic regions in both *P. davidiana* and *P. alba* (the values are shown as mean ± standard deviation).

**Parameters**	**Species**	**Regions diplaying high differentiation**	**Regions diplaying low differentiation**	**Background**
θ_π_	*P. davidiana*	0.0062(±0.0036)^***^	0.0346(±0.0253)^***^	0.0125(±0.0056)
	*P. alba*	0.0089(±0.0045)^***^	0.0359(±0.0151)^***^	0.0159(±0.0023)
Tajima's D	*P. davidiana*	−0.9175(±0.5101)^***^	−0.0650(±0.5126)^***^	−0.2988(±0.5026)
	*P. alba*	−1.5966(±0.3958)^***^	−0.6123(±0.5622)^***^	−1.2113(±0.4562)
Fay&Wu's H	*P. davidiana*	−0.5995(±0.3658)^***^	−0.1169(±0.2569)^***^	−0.3898(±0.2759)
	*P. alba*	−0.5136(±0.3042)^***^	−0.1154(±0.2368)^***^	−0.3125(±0.2032)
r^2^	*P. davidiana*	0.2876(±0.1513)^***^	0.1969(±0.0926)	0.2102(±0.1312)
	*P. alba*	0.2428(±0.1368)^***^	0.1659(±0.0960)^*^	0.1536(±0.1123)
ρ/θ_π_	*P. davidiana*	0.1655(±0.2643)^***^	0.1142(±0.1199)^**^	0.2332(±0.3502)
	*P. alba*	0.2588(±0.3535)^***^	0.2066(±0.2356)^**^	0.5399(±0.5201)
Fixed (%)	*P. davidiana*	0.0565(±0.0315)^***^	~0(±0.0000)^***^	0.0055(±0.0099)
	*P. alba*	0.0431(±0.0291)^***^	~0(±0.0000)^***^	0.0037(±0.0076)
Shared (%)		0.0788(±0.0338)^***^	0.3466(±0.0933)^***^	0.1675(±0.0528)
*F_*ST*_*		0.7305(±0.0410)^***^	0.1125(±0.0179)^***^	0.3806(±0.1615)
d_xy_		0.3036(±0.0025)^***^	0.0422(±0.0188)^***^	0.0252(±0.0121)
RND		0.7261(±0.2365)^***^	0.6523(±0.1887)^***^	0.4998(±0.1501)

Asterisks designate significant differences between the regions displaying exceptionally genetic differentiation and the rest of genomic regions by Mann-Whitney U test (^*^P < 0.05; ^**^P < 1e-4; ^***^P < 2.2e-16).

Nevertheless, long-term balancing selection characteristics are exhibited by regions of low differentiation (Charlesworth et al., 1995). For instance, less differentiation was exhibited by the dxy and RND values of the regions with low differentiation between both populations in comparison to the regions manifesting high differentiation (Figures 4A,B), both *P. alba* and *P. davidiana* populations exhibiting a notably high level of polymorphism (π) (Figures 5A,B). Frequently appearing intermediate-frequency alleles were represented by the higher Tajima's D and Fay & Wu's H values (Figures 5C–F), with LD levels lower in comparison to the corresponding levels in the regions with high differentiation (Figures 5G,H), possibly under the influence of recombination (Lee et al., 2011). In the low differentiated regions, the ratio of inter-specific shared polymorphisms was rather high (Figure 4C) whereas the proportion of fixed differences was found to be trivial in both the *P. alba* and *P. davidiana* populations (Figures 5K,L).

### Influence of the Rate of Recombination on Genome Differentiation

Another important factor affecting the differentiation of the genome is the rate of recombination. Recombination rates were calculated using FastEPRR software (ρ = 4*N*_*e*_c) over a 10,000 bp window size. Since ρ = 4*N*_*e*_c, a decline in *N*_*e*_ in an area linked to selection decreases the local estimate of ρ. In an attempt to get rid of this impact, the influence of recombination rate upon genomic differentiation was evaluated by estimating ρ/θ_π_ in regions with high or low differentiation. Particularly, a notable negative correlation was found between the rate of recombination and *F*_*ST*._Highly differentiated genomic regions exhibited a very low rate of recombination, whereas a low differentiated genomic region had a much higher rate of recombination (Figures 5I,J). This outcome suggests that in the *P. alba* and *P. davidiana* populations, the rate of recombination has an indispensable role in the genomic differentiation process.

### Genes Under Selection

**Figure 6:**
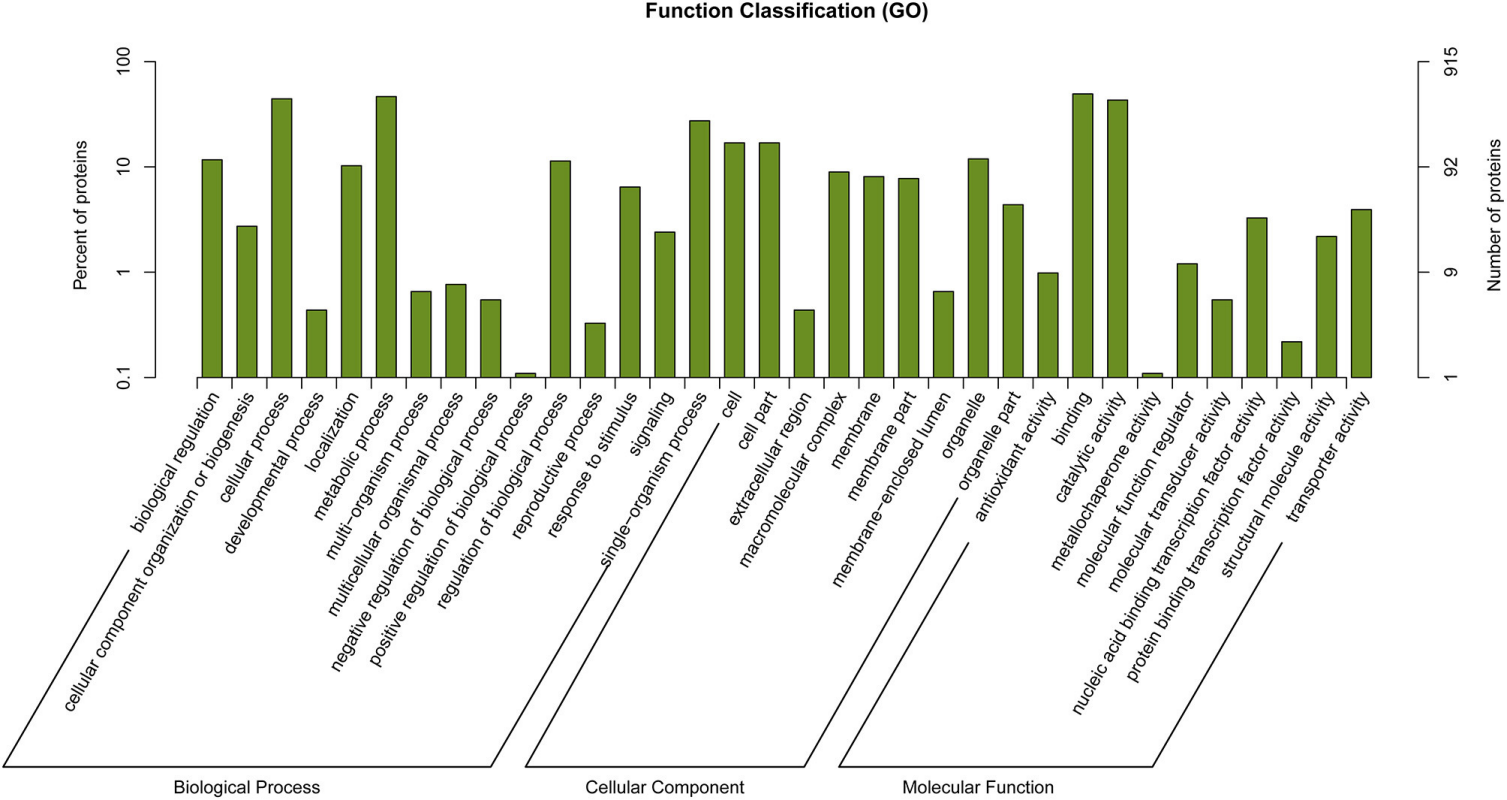
GO enrichment analysis in highly differentiated regions between *P. davidiana* and *P. alba*.

**Figure 7:**
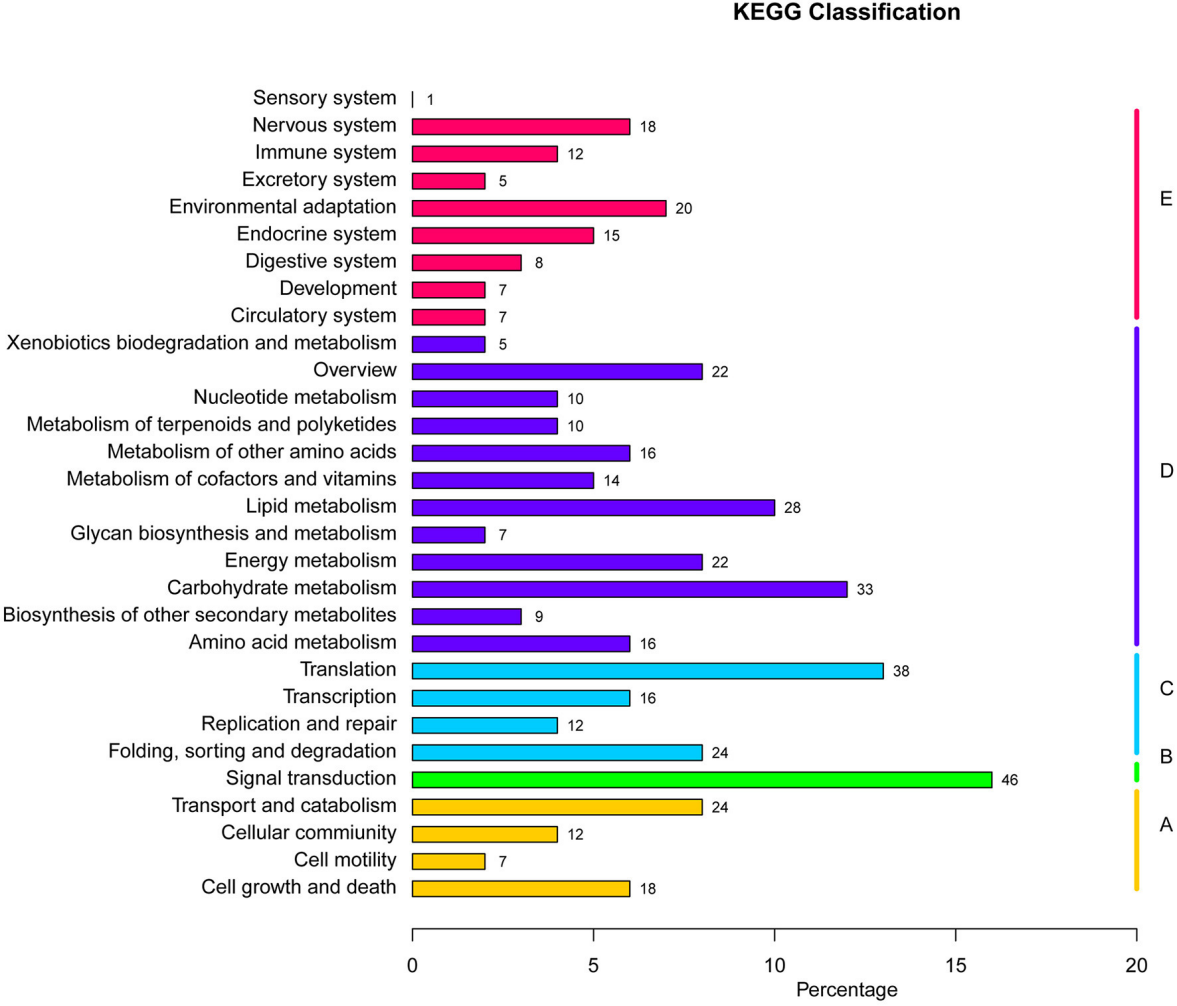
KEGG pathways enrichment analysis in highly differentiated regions between *P. davidiana* and *P. alba*.

Functional annotations on regions manifesting high differentiation in *P. davidiana* and *P. alba* were performed using annotation of the reference genome of *P.alba*. The present study identified 556 selected genes in total. The differential enrichment of candidate genes was investigated using gene ontology (GO), and 34 significantly overrepresented GO terms were found for genes located in zones with high genetic differentiation. The majority of these GO categories were associated with stimulus-response, catalytic activity, and positive regulation of biological processes (Figure 6). The KEGG pathway enrichment method (using the KOBAS system) also found that functional genes related to cell growth and death, signal transduction, environmental adaptation, and immune system functions were all significantly enriched (Figure 7). Thus, the results demonstrate that the functional genes associated with environmental adaptation are majorly responsible for the differentiation in *P. davidiana* and *P. alba* populations. Presumably, genes with similar functions were subjected to strong selection pressure, resulting in significant evidence of increased genetic differentiation and selection in *P. davidiana* as compared to its sister species, *P. alba*, however more functional studies are needed to confirm this. In this work, genes related to the adaptation to environmental factors or photosynthesis have been identified. Such genes could be used as contenders in higher-level studies of the mechanisms governing important features, and they could be useful in preserving the populus in the face of a variety of challenges.

## Discussion

We use a population genomic approach to resolve the evolutionary histories of two widespread and closely related forest tree species, and to highlight how genome-wide patterns of differentiation have been influenced by a variety of evolutionary processes. We estimated dxy and *F*_*ST*_ values across the genome and discovered distinct genetic differentiation between *P. alba* and *P. davidiana*. In order to understand how multiple diversified evolutionary forces driving the demographic history and differentiation of the two distinct populations, a whole-genome resequencing method was applied on the basis of a large count of impartial SNPs dispersed through the entire genome. The findings suggest that Quaternary climate change leading to geographic isolation may have aided in the initial divergence of *P. alba* and *P. davidiana*, and that natural selection plays an essential role in preserving species distinctiveness after gene flow.

### Demographic History of the Two Aspen Species

Demographic histories of *P. davidiana* and *P. alba* were inferred by employing a coalescent simulation-based approach in fastsimcoal 2.6.1 (Excoffier et al., 2013). According to the best-fitting demographic model conducted from Fastsimcoal2, the early divergence of two species was estimated to initialize ~802,365 years ago (Figure 2A), the glaciation events and geological uplift observed for the Qinghai–Tibetan Plateau (QTP) in the MLP coincide with the time of differentiation (Zheng et al., 2002). About 0.6–1.2 Ma on the QTP, the Kun-Huang diastrophisms took place (Hsü, 1982). At the turn of the early–late and MLP, a powerful regional uplift took place around the Yellow River drainage, leading to the glaciation of the QTP (Zheng et al., 2002). That precisely was the Naynayxungla Glaciation, which also took place around 0.8 Ma (Zheng et al., 2002). Amidst this cold period, a large part of the QTP was covered with ice sheets which could potentially fragment the habitats belonging to ancestral populations, thereby limiting gene flow among populations. When the Naynayxungla Glaciation was at its peak, the ice sheet covered a region that was five to seven times greater than what it covers now (Wu et al., 2001). When the Kun-Huang diastrophisms and the Naynayxungla Glaciation arrived, the climate in China did not allow warm adapted plants to grow and the subtropical vegetation moved from north to south of Qinling Mountains (Zheng et al., 2002). Because gene flow was practically impeded by the species' geographical isolation, a large-scale succession of plant communities occurred, and geological uplift and glacier events may have caused the differentiation of the ancestors of *P. davidiana* and *P. alba* in China. The divergence time was consistent with *Cupressus chengiana* on QTP (Li et al., 2020), which diverged at 0.8 Ma and was influenced by the Kun-Huang diastrophisms and the Naynayxungla Glaciation.

Geographic isolation between various populations was an obvious consequence of regional uplift and quaternary climate fluctuations (Han et al., 2017). The geographic barriers theory was validated by our research, and the discontinuous distribution pattern of *P. davidiana* and *P. alba* could have been the result of geographic barriers where their ancestral populations started to differentiate due to vicariance. Due to previous geological events such as the Kun-Huang diastrophism and the Naynayxungla Glaciation, as well as significant climate oscillations, geographic barriers confined *P. davidiana* and *P. alba* into separate continental regions, effectively preventing gene flow between the two species. For instance, we discovered obvious genetic differentiation among both populations under consideration, the gene flow between *P. davidiana* and *P. alba* was considerably low (3.97 × 10^−8^ and 9.16 × 10^−7^) and the *F*_*ST*_ values corresponding to *P. davidiana* and *P. alba* were around 0.2988 (Table 5) and (Figure 2A), which further proved that the existence of geographic barriers significantly obstructed the gene flow between *P. davidiana* and *P. alba* to rather insignificant levels. Moreover, for *P. davidiana* and *P. alba*, the distribution of ancestral population could have further fragmented due to Naynayxungla Glaciation and Kun-Huang diastrophism (for instance, the existence of glacial refugia), thus driving the divergence of species. Gene flow had been impeded among populations due to geographical isolation (Hancock and Bergelson, 2011). Simultaneously, owing to the differing selection pressures of various populations, the isolated populations slowly gathered variation, leading to the differentiation between *P. alba and P. davidiana*. Earlier studies have reported a noteworthy impact on the biodiversity of the QTP as a result of Quaternary climatic oscillation (Liu and Harada, 2014), which led to the current species diverging in interspecific (Xu et al., 2019) and intraspecific (Guangpeng et al., 2017) fashion. Therefore, Quaternary climatic changes may have cast the primary influence on the divergence that initially took place among *P. davidiana* and *P. alba*. Our findings for these two aspen species support an allopatric model of speciation, given their current geographic isolation, extraordinarily low rates of gene flow, and disjunct distribution (Morjan and Rieseberg, 2015).

**Table 5:** Mean (±standard deviation) values of population genomic statistics (θ_π_, θ_w_, H_E_, Tajima's D, ρ, *F*_ST_ and dxy) comparisons between *P. davidiana* and *P. alba* population.

**Species**	**θ_π_**	**θ_w_**	**H_E_**	**Tajima's D**	**ρ**	***F_ST_***	**d_xy_**
*Populus davidiana*	0.0089	0.0156	0.0125	0.13	1.22	0.2988	0.2658
*Populus alba*	0.0095	0.0175	0.0235	−0.06	2.36		

PSMC indicated that a significantly long-term bottleneck was encountered by these two aspens post divergence, with population expansion initiating after the conclusion of the last glacial maximum (LGM) that took place around 20,000 years ago (Figure 2B). A variety of other forest trees found in Eurasia corroborate well with this demographic data (Hewitt, 2000, 2004).

### Heterogeneous Genomic Differentiation of *P. alba* and *P. davidiana*

Consistent with the expectation for allopatric speciation, loss of gene flow, and stochastic genetic drift resulted in the build-up of interspecific differentiation (Chen et al., 2018). Quite a few regions of genomic differentiation were detected between the two populations. Though neutral processes can effectively explain the majority of these in the two populations (Strasburg et al., 2012), natural selection has had a significant impact on a few outlier regions (Nielsen, 2005). If natural selection has truly been one of the dominant evolutionary forces shaping patterns of genetic differentiation between the two species, regions of low recombination would be expected to show increased *F*_*ST*_ values (Noor and Bennett, 2009), because neutral variation is most often removed by a natural selection including background selection and selective sweeps, particularly in areas having a very low recombination rate (Begun et al., 2007). As a consequence, relative measures of divergence (e.g., *F*_*ST*_) that rely on within-species diversity are expected to be higher in regions with restricted recombination (Noor and Bennett, 2009; Nachman and Payseur, 2012). A notable negative correlation between recombination rate (ρ) and *F*_*ST*_ was found in the populations of both *P. alba* (Figure 5I) and *P. davidiana* which is in good agreement with the above observations (Figure 5J) (Keinan and Clark, 2012). Consequently, our results indicate that ρ and linked selection were key factors affecting genomic differentiation among the populations of *P. davidiana* and *P. alba* (Turner et al., 2005; Cruickshank and Hahn, 2014).

Rather than being physically clustered into just a few large, discrete genomic “islands” as expected when species diverge in the presence of gene flow, differentiation islands in our study system are distributed throughout the genome, being narrowly defined and mostly located in regions with substantially suppressed recombination (Turner et al., 2005; Cruickshank and Hahn, 2014). Neutral variation linked to a deleterious mutation is lost through background selection, and neutral variation linked to a positive mutation is fixed by the genetic hitchhiking of positive selection (Turner et al., 2005; Noor and Bennett, 2009; Cruickshank and Hahn, 2014). Hence, several population genetic parameters were evaluated to understand how the whole genome differentiation in *P. davidiana* and *P. alba* populations was driven by diverse evolutionary forces and how genomic variation took place during the sequence of population differentiation (Figure 6). We found that significant positive selection characteristics were exhibited in the two populations by the region having high differentiation (Nielsen, 2005). Both *P. davidiana* and *P. alba* populations, for example, manifest a very low level of polymorphism (π) (Figures 5A,B). The powerful signals of linkage disequilibrium, higher RND, and dxy values were a more obvious feature of the regions having high differentiation (Figures 4A,B and Figures 5G,H), indicating absolute interspecific divergence. It has also been highlighted by quite a few recent studies that in a selective sweep model, genetic variants associated with useful mutations are exposed to positive selection and attain a high frequency by hitchhiking along (Kaplan et al., 1989; Mei et al., 2018). Selection due to factors such as local ecological adaptation, particularly in the absence of gene flow, can indirectly inflate *F*_*ST*_ by leading to reduced within-population diversity (Cruickshank and Hahn, 2014). Therefore, although a contribution of background selection to the observed patterns cannot be completely discounted, the independent action of positive selection in both *P. davidiana* and *P. alba* is expected to be the dominant driver for the evolution of reduced diversity and increased differentiation in most islands of differentiation. Although genetic diversity underwent a significant decrease under the influence of positive selection, there was an increase in interspecific differentiation. However, because assessing variance in these locations with high differentiation and low genetic diversity is difficult, extra caution should be exercised when interpreting the functional features of the overrepresented genes identified in this example. As a result, more research into these functional genes is required in order to understand how forest tree species respond to the stimulus of climate change throughout adaptive evolution.

While the highly differentiated regions exhibited features of positive selection, the regions with low differentiation in both populations were also identified to manifest long-term balancing selection (Charlesworth et al., 1995). Long-term balancing selection supports maintaining beneficial polymorphisms for quite many generations as compared to positive purifying and selection, resulting in regions of the genome with reduced *F*_*ST*_ and increased genetic diversity (Guerrero and Hahn, 2017). In this study, in comparison to the highly differentiated regions, absolute interspecific divergence (dxy and RND values) was found to be lower (Figures 4A,B). Both *P. davidiana* and *P. alba* populations had a notably high genetic diversity (π) (Figures 5A,B). Balancing selection may be identified due to its influence upon neutral sites lying nearby when the same alleles persist for a long time. The population genetics of balancing selection shows that, despite maintaining diversity at the selected sites (usually distinct amino acids), there is a concurrent rise in diversity at closely connected neutral sites (Mei et al., 2018). The frequent appearance of intermediate-frequency alleles is indicated by higher Tajima's D and Fay & Wu's H values (Figure 5F), with levels of LD lower in comparison to the highly differentiated regions, which may have been impacted by recombination (Lee et al., 2011). The proportion of inter-specific shared polymorphisms was found to be higher in low differentiated regions (Figure 5C) in both the *P. davidiana* and *P. alba* populations, the proportion of fixed differences was found to negligible (Figures 5K,L). While selection reduces diversity at selected loci and linked gene regions in general, balancing selection frequently works to maintain diversity at such loci, allowing distinct beneficial variations to be retained over long periods (Begun et al., 2007). Our results, thus, suggest that long-term balancing selection, presumably regulated by “recycling polymorphism” (Holub, 2001) or “trench warfare” (Stahl et al., 1999) of co-evolutionary interaction between natural enemies and hosts, may have caused an increase in genetic diversity over extended periods (Salvaudon et al., 2008).

## Conclusions

Here we provide insights into the speciation and recent evolutionary histories of two closely related forest tree species, *P. davidiana* and *P. alba*. The genetic differentiation between the two species was found to be obvious, with an *F*_*ST*_ value of 0.2988 for *P. davidiana* and *P. alba*. Coalescent simulations suggest that the divergence of the two species occurred in the middle Pleistocene, with only minimal gene flow after the divergence. Following divergence, the two species encountered a significant long-term bottleneck, with population increase beginning roughly 20,000 years ago, at the end of the last glacial maximum. Though neutral processes explain the vast majority of regions of genomic differentiation between the two species, a few outlier regions that are noticeably influenced by natural selection have also been investigated. The highly differentiated regions of both species showed substantial positive selection characteristics, and long-term balancing selection was also observed in the regions with limited differentiation in both species. Our results point out that Natural selection and Quaternary climate changes both played a role in the divergence of the *P. davidiana* and *P. alba* populations.

## Data Availability Statement

The data presented in the study are deposited in the Genome Sequence Archive repository (http://bigd.big.ac.cn/gsa), accession number CRA003302 and CRA001674.

## Author Contributions

ZH performed the experiments and wrote the study. AL designed the research. All authors contributed to the article and approved the submitted version.

## Funding

Financial support for this research was provided by the Fundamental Research Funds of Sichuan Provincial Science Program Project (22NSFSC3363), China West Normal University (Grant No. 20B007 and 19E044), the Open Fund of Ecological Security and Protection Key Laboratory of Sichuan Province, Mianyang Normal University (Grant No. ESP2004), and the Open Fund of MOE Key Laboratory of Biodiversity and Ecology Engineering, Beijing Normal University (Grant No. K202001).

## Conflict of Interest

The authors declare that the research was conducted in the absence of any commercial or financial relationships that could be construed as a potential conflict of interest.

## Publisher's Note

All claims expressed in this article are solely those of the authors and do not necessarily represent those of their affiliated organizations, or those of the publisher, the editors and the reviewers. Any product that may be evaluated in this article, or claim that may be made by its manufacturer, is not guaranteed or endorsed by the publisher.
