# Supplementary material for: Genomic Differentiation and Demographic Histories of Two Closely Related Salicaceae Species
Source: Front Plant Sci. 2022 Jun 7;13:911467. doi: 10.3389/fpls.2022.911467 (PMC9210983; doi:10.3389/fpls.2022.911467)
Supplement: Supplementary file 1 [file Data_Sheet_1.zip › Figure S1.docx]

**Figure S1.** Tested demographic models. Model1, isolation of two species without gene flow; model2, isolation of two species with asymmetric gene flow; model3, isolation of two species with exponential population size change in *P. alba* and stepwise population size change in *P. davidiana*, no gene flow; model4, isolation of two species with exponential population size change in *P.alba* and stepwise population size change in *P. davidiana*, with asymmetric gene flow; model5, isolation of two species with exponential population size changes in both species, no gene flow; model6, isolation of two species with exponential population size changes in both species, with asymmetric gene flow; model7, isolation of two species with stepwise population size changes in both species, asymmetric gene flow in the early stage of species divergence until the time of TISO, no gene flow afterwards; model8, isolation of two species with stepwise population size changes in both species, no gene flow in the early stage of species divergence until the time of TISO, asymmetric gene flow afterwards; model9-model11, isolation of two species with two steps of population size changes in both species, both species experienced stepwise population size changes until the time of TISO, afterwards, *P.alba* experienced exponential population size change, and *P. davidiana* experienced another stepwise change, the difference between models is the occurrence and the time of gene flow between species; model12-model14, isolation of two species with two steps of population size changes in both species, both species experienced stepwise population size changes until the time of TISO, afterwards, both species experienced exponential population size changes, the difference between models is the occurrence and the time of gene flow between species ; model 15-model18, isolation of two species with three steps of stepwise population size changes in both species, the difference between models is the occurrence and the time of gene flow between species.
