# Supplementary material for: Genomic Differentiation and Demographic Histories of Two Closely Related Salicaceae Species
Source: Front Plant Sci. 2022 Jun 7;13:911467. doi: 10.3389/fpls.2022.911467 (PMC9210983; doi:10.3389/fpls.2022.911467)
Supplement: Supplementary file 1 [file Data_Sheet_1.zip › Table S1.docx]

**Table S1.** *F*_ST_ and dxy comparisons between *P. davidiana*, *P. alba*, *P. tremula* and *P. tremuloides* populations

| Parameters | *P. davidiana-P. alba* | *P. davidiana-P. tremula* | *P. davidiana-P. tremuloides* | *P. alba-P. tremula* | *P.alba-P. tremuloides* |
| --- | --- | --- | --- | --- | --- |
| *F*_ST_ | 0.2988 | 0.3620 | 0.2856 | 0.2896 | 0.3125 |
| dxy | 0.2658 | 0.2498 | 0.2588 | 0.2468 | 0.2725 |
