# Supplementary figures and images for: Genomic Differentiation and Demographic Histories of Two Closely Related Salicaceae Species
Source: Front Plant Sci. 2022 Jun 7;13:911467. doi: 10.3389/fpls.2022.911467 (PMC9210983; doi:10.3389/fpls.2022.911467)

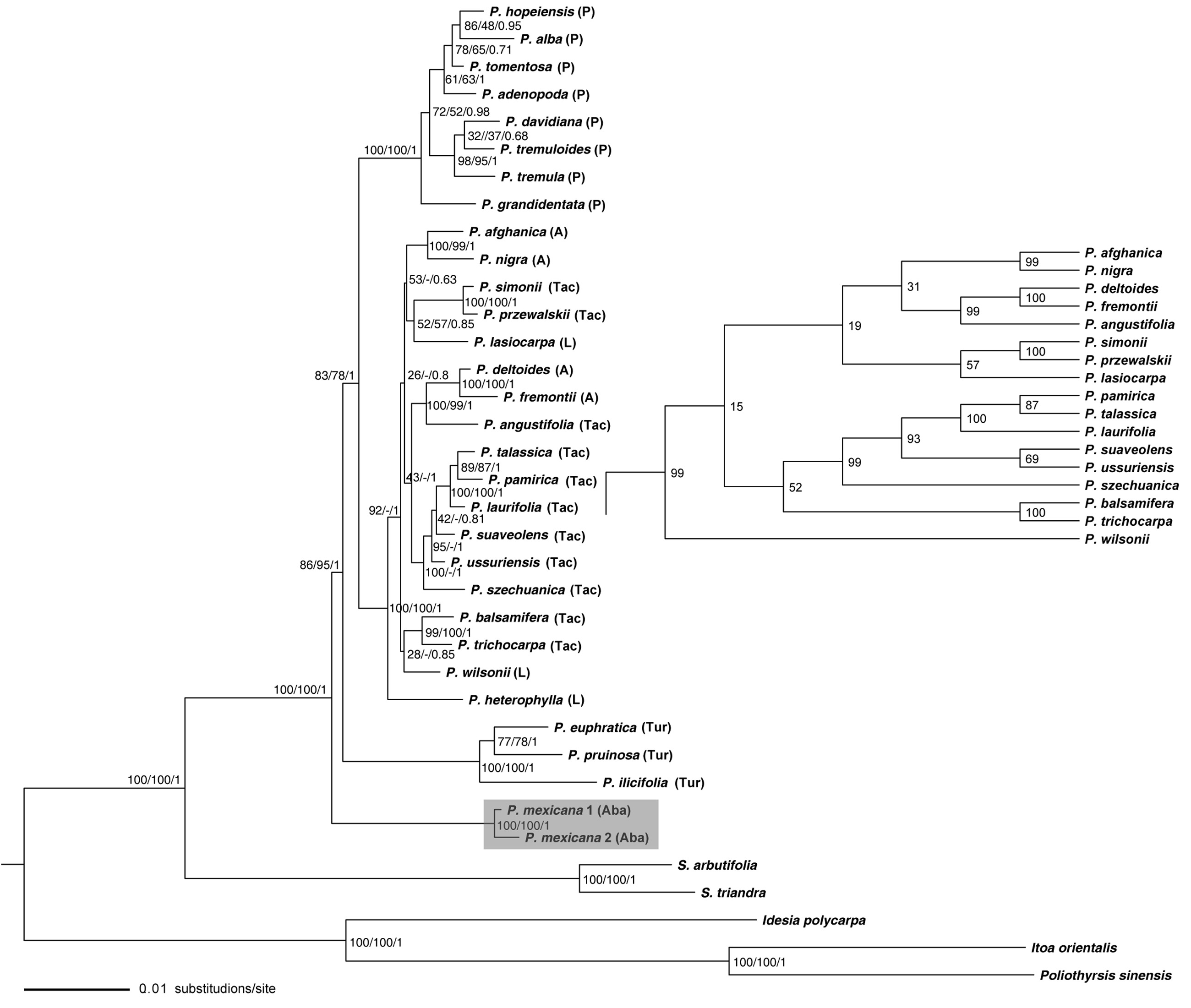

Supplement: Supplementary file 1 [file Data_Sheet_1.zip › Image S1.JPEG]

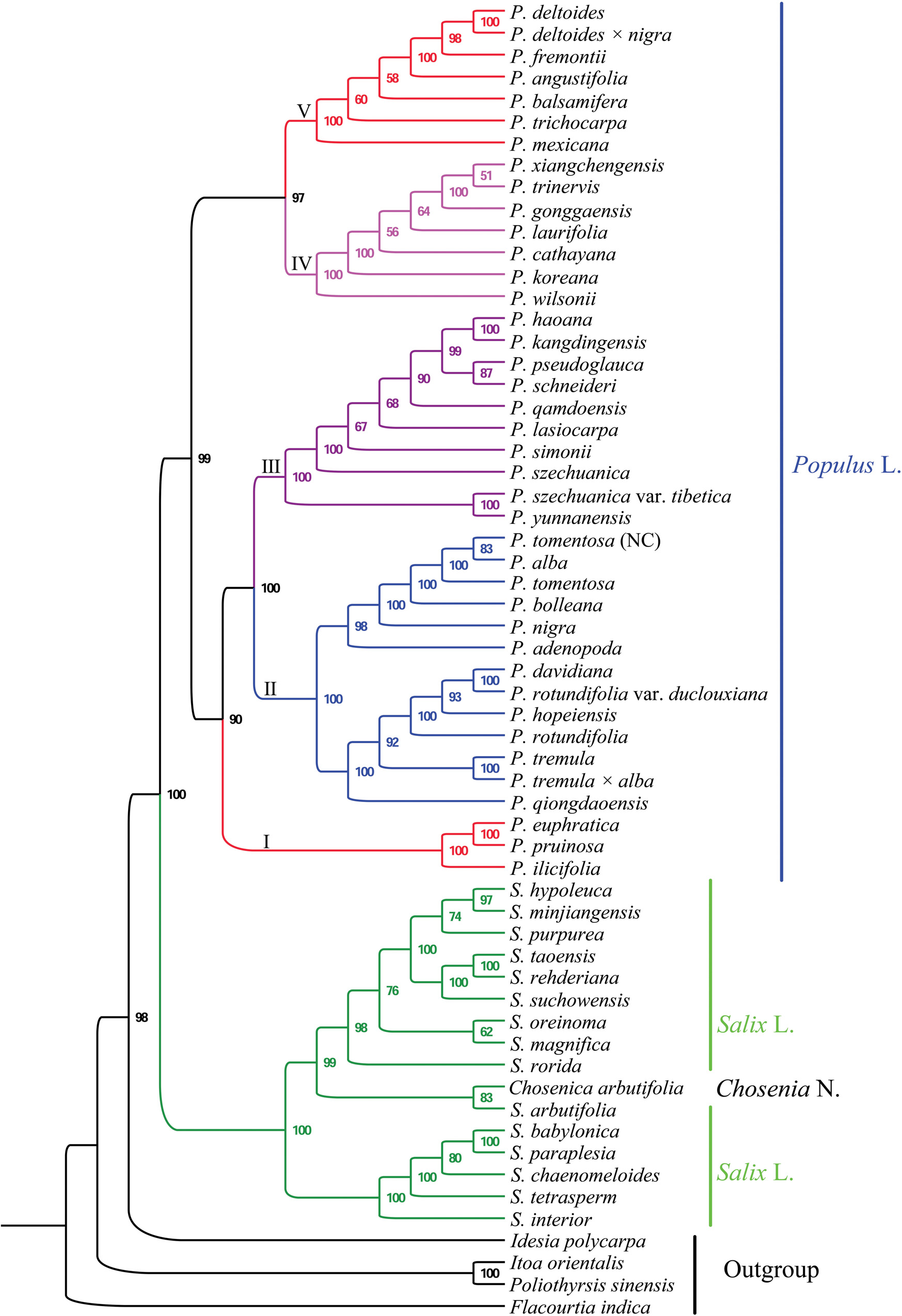

Supplement: Supplementary file 1 [file Data_Sheet_1.zip › Image S2.JPEG]
